# Working Conditions for Burns Resident Doctors—Better Now than Ever?

**DOI:** 10.3390/ebj5040029

**Published:** 2024-09-25

**Authors:** Grant Coleman, Toby Austin, James F. Forrest, Sarah E. Bache

**Affiliations:** Department of Burns and Plastic Surgery, Queen Elizabeth Hospital, Birmingham B15 2TH, UK

**Keywords:** junior doctors, job satisfaction, burns, workload, historical article, work–life balance, surgical training, inter-generational attitudes

## Abstract

Background: The work and life of a resident (or “junior”) doctor has changed dramatically over the past 50 years. Descriptions of historic working conditions are usually anecdotal and tinted with nostalgia, but do today’s burns and plastic surgery doctors feel working conditions have improved or declined over the last 50 years, and does this have an impact on recruitment and retention? Methods: An interview was conducted with a retired surgeon who, in 1970, worked as a house surgeon (Year 2 doctor equivalent) in a burns unit for the pioneering burn surgeon Mr. Douglas MacGregor Jackson. This was coupled with a literature review to objectively assess working conditions in that period for doctors. The information generated from this produced a poster summarizing the key differences between these periods. This was presented to the current medical work force, and a survey was conducted to determine their preferences for working conditions. Results: The questionnaire was completed by 68 doctors of mixed grades and backgrounds. The majority of respondents (60%) would choose to work in today’s burns centres. There was a significant difference between the mean age of respondents’ preference of working conditions in 1970 (37 years) and those preferring today (31 years) (*p* = 0.035). Conclusions: Multiple changes in the working conditions and the management of burns patients were identified. The majority of those who were asked consider today’s working conditions to be better than those of the past. However, more senior clinicians tended to prefer the conditions of 1970 over the present day, suggesting a generational shift in opinion.

## 1. Introduction

“The past is a foreign country: They do things differently there.” [1].

Whilst it is universally acknowledged that the work and life of a resident (or “junior”) doctor has changed significantly over the past 50 years, the precise details of this past are often piecemeal, ethereal or half remembered. Handed down from surgical generation to generation via family or friends, and often with the prefix “back in my day…”. Was the life of a resident doctor half a century ago, working in the same department, objectively better or worse than today? One view is that in the “good old days” patient care was simply better: Better continuity of care and familiarity with one’s patients; vastly improved exposure to surgical problems and increased breadth of practice; a greater feeling of being part of a team, with the “firm” structure and close bonds of doctors who often lived on site and frequented the doctors’ mess. Many believe that doctors were more respected (and possibly more respectable), and although you worked hard, you played hard. Conversely, the very reasons for the introduction of measures such as enforced rest periods and shift-based work patterns are the proven dangers of over-worked, under-rested doctors and a desire to attract a more diverse workforce. Which of these viewpoints is correct, and does it matter whom you ask?

The life of a resident doctor (equivalent to Senior House Officer, SHO or Foundation Year 2, FY2) in one UK-based burns service in 1970 was explored through a first-hand account with a resident doctor of that time. This information was fact checked with a literature review. A contrast was then made with life today in the same unit, and information presented to medical staff within the burns centre in the form of a poster. The question was posed: “Would you rather be a burns SHO in 1970 or now?” By exploring the working conditions of the past, and assessing current burns clinicians’ attitudes to the changing workplace, we aimed to glean greater insight into our unique history, but also to explore the present attitudes toward work, and perhaps where the direction of travel may be for future working conditions.

## 2. Methods

### 2.1. Setting

**Figure 1:**
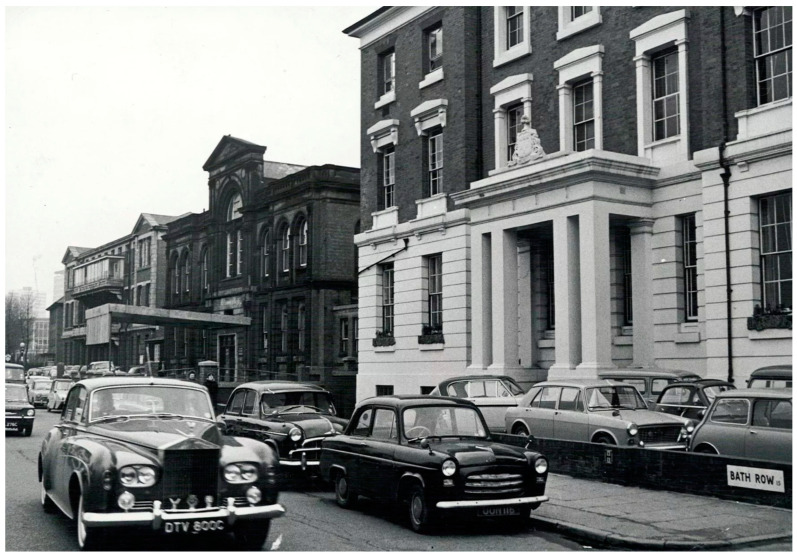
Entrance to “The Acci”, Birmingham, Circa 1970.

Today, the Queen Elizabeth Hospital, Birmingham, is home to the Midlands Burn Care Network’s regional burn centre. Comprising a 15-bed adult burns centre, a burns theatre and a general critical care unit, which can house up to six further critically ill burns patients. Children are treated at the nearby Birmingham Children’s Hospital in the regional children’s burns centre. This comprises seven inpatient beds and an adjacent burns theatre and critical care unit to house ventilated patients. Both services have operating lists five days a week. Seven full-time consultants cover across both sites, with resident doctor cover provided by a mixture of burns fellows, plastics registrars and FY2/SHOs. The new Queen Elizabeth Hospital was built in 2010, but the home of the burns centre in 1970 for both adults and children was the Birmingham Accident Hospital (BAH) (Figure 1).

The BAH (commonly referred to as “The Acci”) was one of the world’s first trauma centres. The Medical Research Council (MRC) Burns and Industrial Injuries Unit within the BAH was formed in 1944, before the creation of the NHS in 1948. It has a rich history, which has been documented with written and photographic archives [2]. The model of centralised burns care established here by the centre’s founder Mr. Douglas Jackson and colleagues, including Mr. Jack Cason in the 1960s and 70s, who was key to the design of the national burns service in place today. Mr. Jackson was an early advocate of the team approach to modern burns management, and pioneered excision and grafting, beginning in 1954 with small burns and gradually increasing up to 30% Total Burn Surface Area (%TBSA). Prior to this, the process of expectant management and allowing burns to “slough and separate” had been largely practiced [3,4].

He published several important papers, including describing the zones of burn injury in 1953, which forms the basis for current understanding of the pathophysiology of burn wound progression and the potential to limit or increase harm caused by the initial insult to the zone of stasis. He described diagnosing the depth of burn injury using methods including the pinprick test [5,6]. In 1972, he authored “Tangential excision and grafting of burns—The method, and report of 50 consecutive cases” [7]. The interviewee remembers Mr. Jackson travelling to the United States having been invited to present his work to a trans-Atlantic audience, as someone at the forefront of one of the most pivotal changes in the management of burns in the last century.

The burns unit established by Jackson moved in 1993 from BAH to Selly Oak, before finally moving to its current sites at the Queen Elizabeth Hospital and Birmingham Children’s Hospital.

During the lifespan of the burns service in Birmingham, there have been massive changes in the scientific understanding and medical practice of burns care. There has also been much wider political, economic and social change within the nation that it serves. These factors mean that the life of a resident doctor working within the burns unit has evolved, with wide-ranging differences in working hours and conditions, dress, pay and living standards, in addition to the care administered to burns patients.

### 2.2. The Interview Process

A semi-structured interview was carried out with a retired surgeon by a current SHO/FY2 doctor. A general discussion was followed by closed questions designed to ascertain in detail what an average working week looked like. Examples are given below. Details were verified, if possible, with existing records at the time, and comparisons were made between the experience of the present day SHO/FY2 (Table 1).

Example interview questions:

1. Can you describe your job and work pattern?
2. How did you carry out dressing changes?
3. Did you operate in theatre?
4. What were the main causes of burns you saw?
5. How were major burns treated?
6. Did you ever use cadaveric or animal skin?
7. How would you be notified if a major burn arrived?

### 2.3. Poster Dissemination and Survey

**Figure 2:**
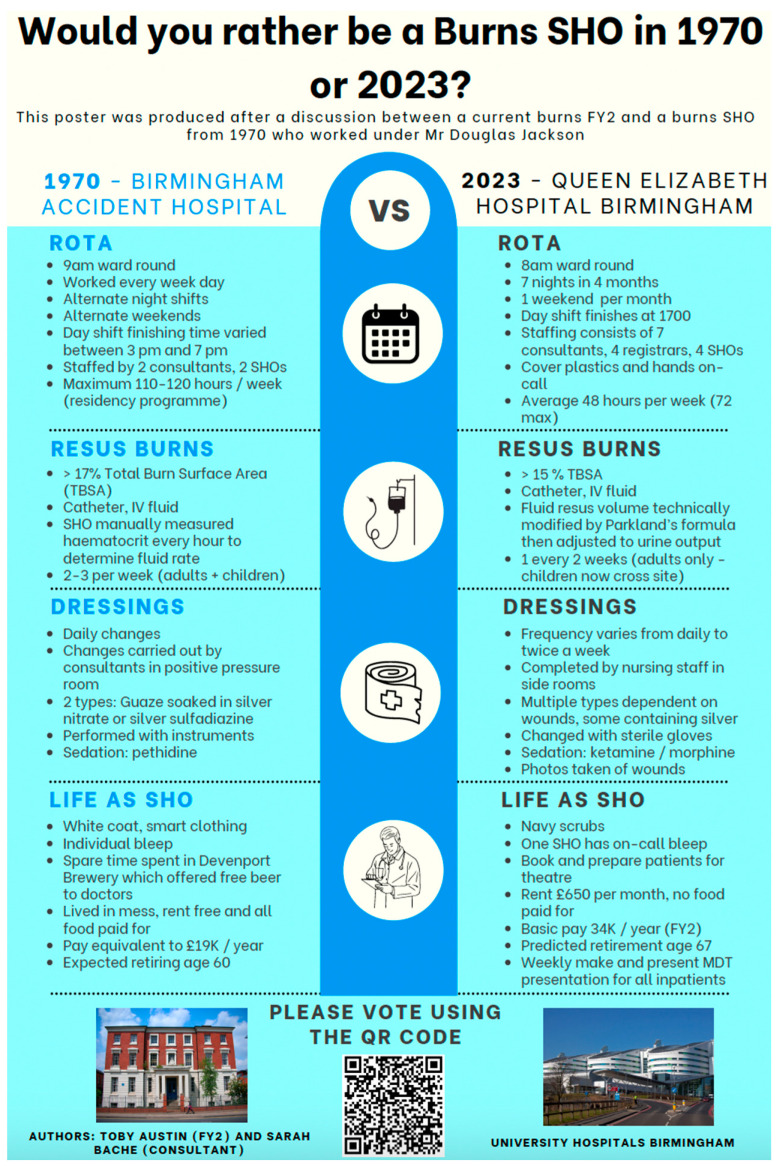
Poster presented to survey correspondents.

The key points raised in the interview were assessed for historical accuracy by performing a literature search, as detailed below. These data were then summarised in a poster (Figure 2). This was displayed in communal areas within the burns centre, and disseminated to all training and consultant level doctors working in the plastic surgery department via group communications platforms (E-mail and WhatsApp). The poster was linked to a QR code to access a voluntary questionnaire from a free survey collection tool (Survey Monkey).

Survey questions included age, training grade and career length, as well as time working as a burns SHO-level doctor. The key question posed by the survey was, after considering all the information, “Would you rather be an SHO in 1970 or 2023?” Responses were excluded if duplicated, incomplete or from non-doctors. Data from the survey were gathered using Microsoft Excel, and statistical analysis was performed with IBM SPSS version 29.0.2.0. Student’s unpaired T-test was performed to demonstrate if there was a difference in age between those who voted for each group. Significance was set at *p* < 0.05.

## 3. Results

### 3.1. The Interview

The interview begins with the lament: “The world has b****y changed…”. In 1970, the world did indeed look very different: just the year prior, man had first walked on the moon and Concorde took its first test flight. In early 1970, the interviewee, then in his 20s, began his three month burns rotation as one of two “house surgeons” for Mr. Douglas MacGregor Jackson and Mr. Jack Cason, the only two appointed consultants at the 30-bed Birmingham Accident Hospital burns unit. With no registrars, he directly reported to them. He describes his average working week.

**Figure 3:**
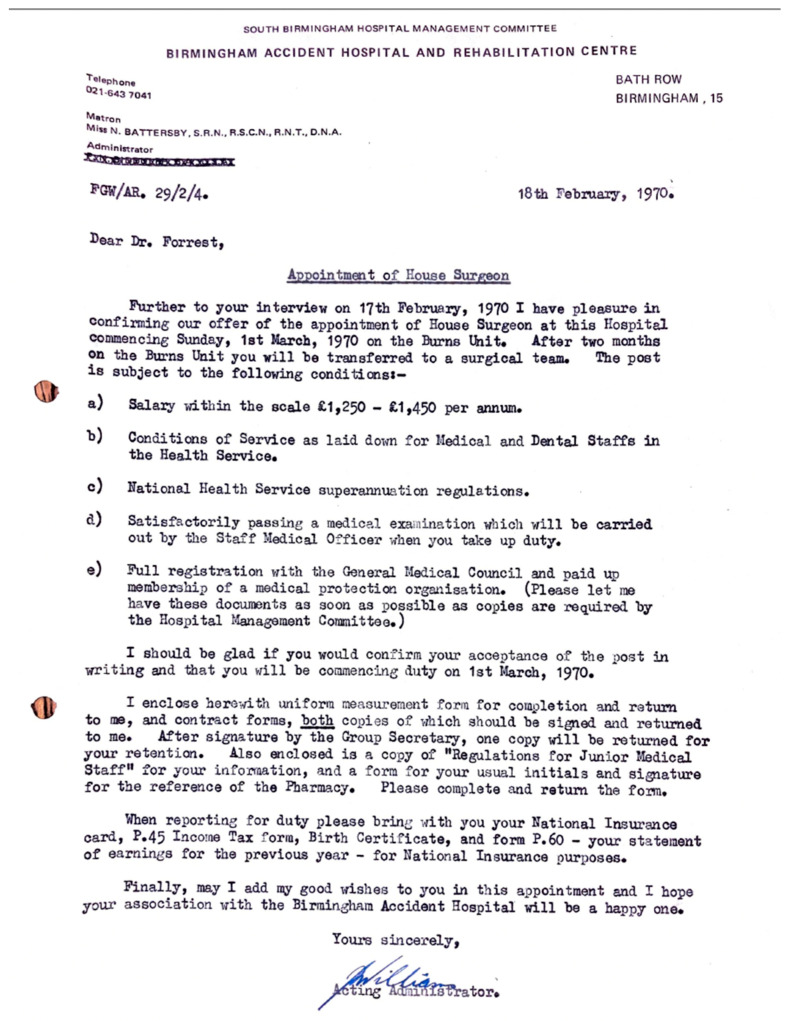
Offer of employment in 1970 with pay scales and conditions of employment.

Waking in the doctor’s accommodation in which, without exception, all resident doctors lived, the interviewee dons his freshly washed and pressed white coat and places his personal pager in his pocket. He is on call today, as he will be for at least eight hours, every working day of this job. He will also be on call every other night and every other weekend, averaging around 106 h a week. The accommodation is, however, provided free of charge for all house officers, in addition to food, drink (notably, unlimited beer, piped directly into the ubiquitous doctors’ mess bar from the next-door brewery) and laundry services. ***“I wore a white coat every single day from my start right up to my retirement in 2007. Fresh daily, identifiable and smart. I resented its demise.”*** With regards to remuneration, the House Officer received GBP 1350 per annum (Figure 3) which he thought at the time was a “princely sum”.

He prepares notes and blood results and awaits with the matron the arrival of the “tall, elegant and always well turned out” Mr. Jackson. ***“We used to start the ward round with a prayer”***, he recalls. Bowing their heads at 9 am, they pray for the health of their patients. He goes on to describe a somewhat familiar pattern of work to today’s burns SHOs: a ward round, bloods, intravenous access, the vigilant monitoring of patients with large burns, silver nitrate-soaked linen or silver sulfadiazine dressing changes, all interrupted occasionally with new referrals from the Accident and Emergency (A & E) department. In contrast to today’s mainly nurse-led service, dressing changes were strictly performed by consultants. Within a positive pressure room, dressings were changed daily with sterile instruments, and intravenous pethidine as sedation. He would attend theatre at least twice a week to act as surgical first assistant.

**Figure 4:**
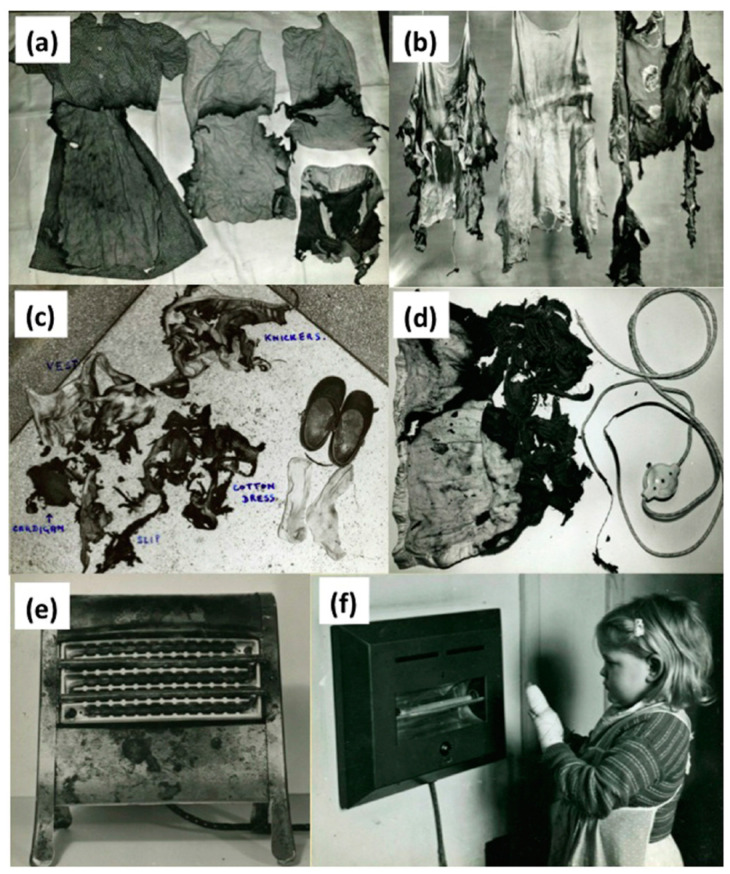
Photographs from the Birmingham Burns unit archive [1] highlighting burns causation, highlighting flammable clothing material (**a**–**c**), faulty electrical items such as an electric blanket (**d**) and exposed heating elements in electric fires (**e**,**f**).

For new referrals, a doctor from A & E would find the on call SHO personally. The interviewee remembers an average of two “large” burns a week. This is possibly more than the present day burns service in Birmingham, although comparisons are difficult due to different thresholds for resus burns, and the separation of adult and paediatric sites. However, the number of major burns is steadily declining despite the city’s population more than doubling in the intervening period, predominantly due to the impact of health and safety legislation [8]. A publication by Jackson from the time describes a different aetiology to that which we see today, with a predominance of burns resulting from coal fires, electric heaters or paraffin heaters (Figure 4) [2]. In comparison, a recent review of the aetiology of patients who presented to the unit over the last decade showed scalds and flame burns being the predominant causes (unpublished data). The interviewee similarly describes the commonly encountered causes of paediatric burn injuries from three bar electric heaters and clothing ignition, mechanisms that are thankfully much less common today.

With regards to life outside of the hospital during this period, the doctors’ mess and bar is where most socialising would occur, with a free bar, not exclusively for those off duty. However, “weekends off” would tend to begin at 3 p.m. on Saturday, with a return to the hospital by 10 p.m. on Sunday, ready for the next working week.

### 3.2. The Comparison

#### 3.2.1. Working Patterns

The interviewee paints a clear picture of his life and work in 1970, and his account may resonate with many who also worked during this period. What is perhaps most striking initially, is how many working hours were normal during this period.

In 1970, his average working week of over 100 h contrasts sharply with today’s SHO/FY2 working a maximum 48 h week. This live-in existence was a tradition for many decades prior to this. Notably, Professor Harrold Ellis recalls in the late 1940s he would have to “…see emergency patients in my pajamas, underneath my white coat” [9]. This workload necessitated living in-house, hence “house officer” or “house surgeon”. The grueling demand of this workload did not go unprotested, with the first “junior” doctor strike conducted by the BMA in 1975, calling for reduced working hours and more equitable pay. Eventually, the subsequent change was facilitated by the introduction of the European Working Time Directive in 1998, which was fully implemented for resident doctors by 2009 [10]. This legislation has facilitated the introduction of protected time “off-duty” around on call shifts, with an aim of safer conditions for doctors and patients.

High intensity of work was, and may still be, believed by many to be offset against greater clinical experience, better comradery with colleagues and seamless continuity in patient care. However, those working as resident doctors in the 1970s retrospectively cited these long hours to have a significant impact on their long-term health, happiness and family [11]. The concept of burnout was not even described until 1974 [12]. By 2017, rates of emotional burnout amongst UK doctors were reported to range from 31 to 54%, with overload and increased hours worked being some of the factors [13].

#### 3.2.2. Finances

The interviewee reports by means of his original letter of appointment (Figure 3) pay of between GBP 1250 and GBP 1450. Taking the median figure of GBP 1350, and using historical inflationary data gathered by the retail price index, this is equivalent to GBP 17,832 (March 2024). The Rt Hon. Mr. Willie Hamilton, Labour Member of Parliament for West Fife in a supplementary debate on the “Junior doctors pay dispute” (history does tend to repeat itself) lays out the pay scales [14]. (Table 2).

Direct salary comparison to the most junior doctor (SHO) working in our burn unit today, however, is challenging. Firstly, the present day most junior member of the burns team is an SHO/FY2, opposed to a house officer, so at least a year senior. Secondly, the financial burden of accommodation and essential bills was non-existent for most resident doctors in 1970. Mess culture meant that unlike today’s SHO in our unit, there was no cost for accommodation, meals, council tax, heating, electricity and commuting. The exact value of all this is difficult to estimate due to individual variation in spending habits. However, using UK average household expenditure on rent bills and food to guide us, this would be in the region of GBP 14,000 to GBP 18,000 per annum. It is worth noting that payment for these goods and services today come after being subjected to income tax, making the true cost of this benefit-in-kind even higher than this figure.

In 1970, the average house price (GBP 3920) was 3.1 times higher than average earnings, which starkly contrasts with today’s house prices being 8.39 times average earnings today, and average prices of GBP 285,431.

Finally, whilst in-depth discussion about the pension system is also outside the scope of this article, the retirement age if you were a resident doctor in 1970 was 60 years old and on a final salary pension. For everyone born after 1978, the current state pension age is 68 years old and pensions are based on average salary. This figure may need to further rise to 71 years old by 2050, according to some independent think tanks in order to support the economic model of retirement in the UK.

#### 3.2.3. Burn Care Advances

In common with all areas of trauma care, burns management has seen great advancements and changes over the last 50 years. This has culminated in an increase of the threshold of expected survivability in major burns. The interviewee recounts 50 years later the full name of a 10-year-old girl with what was considered at the time to be a non-survivable 75% TBSA scald injury. After all this time he becomes visibly moved as he recalls her mother being told by a doctor, shortly after admission, in a brisk matter of fact way, ***“She’s going to die you know”***. With today’s advances, a child with 95% TBSA burns may not be considered futile, let alone 70% TBSA, as described in the interview [15].

There are many elements of burns care that have brought about this change, but perhaps the most significant is the concept of early tangential excision and grafting, reported by Zora Janžekovič in the 1960s [16]. As discussed earlier, Jackson was in many domains of burns research and care a pioneer. Although not an established routine part of burns care in the UK in 1970, as an early proponent of tangential excision and grafting of deep dermal burns, the interviewee recalls Mr. Jackson being invited to America to speak about his experience. This, combined with the famous Jackson’s zone of injury model of burn wounds means the recollections of the doctor working in Birmingham at this time provide a unique insight into the centre at a time of immense change from the traditional practice of allowing the burn to “slough and separate”. Early excision of burn wounds, alongside improved fluid resuscitation, infection control organ support and management of the hypermetabolic response amongst other interventions have all helped to reduce mortality from major burns in the last 50 years significantly [17].

The population that today’s burns centre serves has more than doubled in the intervening 50 years and yet, the number of beds in the unit have halved, as have the number of resuscitation burns presenting to it. Meanwhile, the number of doctors delivering the service today has risen from 4 to 15. From photographic archives that exist, we can see that the problem of burn causation was well known and documented in the 1960s, and was key to the implementation of health and safety legislation that has been the predominant drive for this reduction. The medical complexity, and age of patients presenting with burns have, however increased over time [4,7,15].

## 4. Survey

In total, 68 doctors completed the survey: 25% (*n* = 17) were consultants, 18% (*n* = 12) registrars, 37% SHOs (*n* = 25) and 21% (*n* = 14) foundation year one (FY1) with a broad range of ages from 23 to 60. Over half (54%) of doctors had worked as an SHO/FY2 in a burns unit.

**Figure 5:**
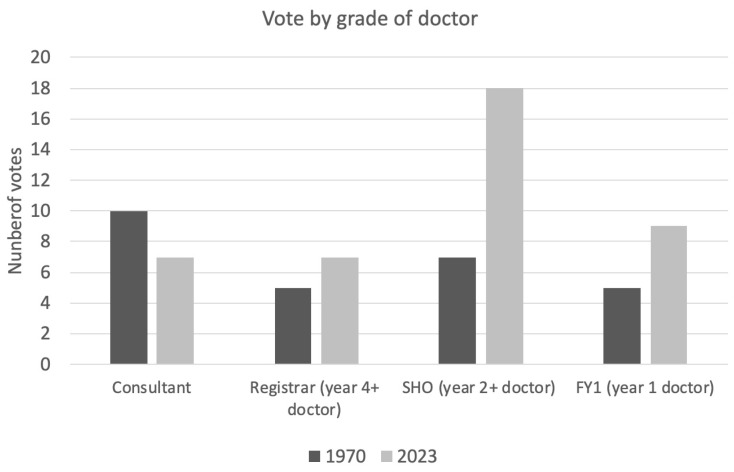
Breakdown of vote for preferred working era by grade of doctor.

In answer to the question ‘would you rather be a burns SHO in 1970 or 2023?’, 40% (*n* = 27) voted for 1970 and 60% (*n* = 41) for 2023. The majority of consultants (59%) voted for 1970. In contrast, the majority of registrars (58%), SHOs (72%) and FY1s (64%) voted for 2023 (Figure 5).

**Figure 6:**
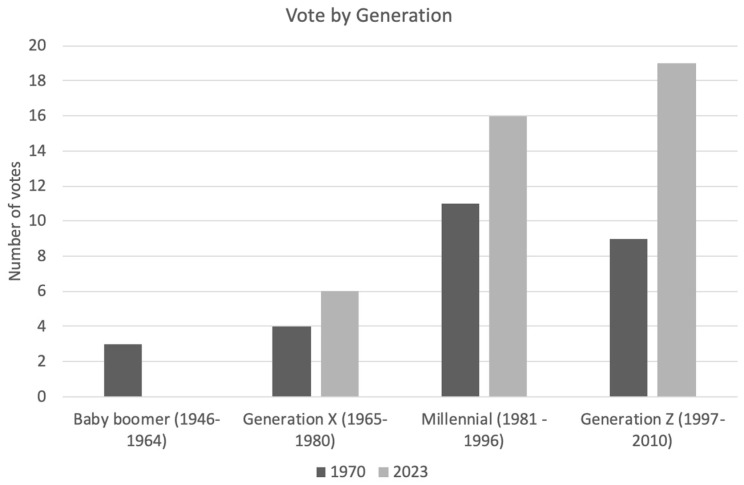
Breakdown of voting by generation, classified by birth year.

The mean age of voter for 1970 vs. 2023 was 37 years versus 31 years, respectively (*p* = 0.035 *unpaired T-test*). Broken down by generation, this is demonstrated in Figure 6. Gender (*p* = 0.164) and prior burns SHO/FY2 experience (*p* = 0.53) were not found to be statistically significant (chi-squared test).

## 5. Discussion

By exploring how burns care has evolved over the last 50 years, told through the eyes of resident doctors within the same burns centre, and contrasting working conditions then and plastic surgeons’ attitudes to the changing workplace, we aimed to pause and reflect on our history and evolution as a specialty. In a climate where recruitment and workforce satisfaction are often negatively reported, we can look with some objectivity to the past and consider if this is justified, or if, as our survey showed, there are many aspects of today’s working practices for a prospective burns surgeon that may be attractive and better than before.

A higher proportion of total votes for today’s working conditions were observed. This is interesting when viewed with the current background in the United Kingdom of issues with workforce motivation, pay disputes and working conditions. Motivating factors behind this could be due to reduced or ‘safer’ working hours, rest days and more time to enjoy activities outside of work. Many, however, would still prefer to work in the conditions of 1970. Perhaps more interesting is the significant age difference observed for this preference.

Inter-generational differences are considered when future-planning by industry. Key domains commonly discussed are the differences in work ethic, life priorities and expectations between generations [18]. Baby Boomers (1946–1964) are described as valuing hard work, loyalty to their employer and traditional career advancement with a strong emphasis on job security. Generation X (1965–1980) tends to prioritise a balance between work and personal life, valuing flexibility and independence. Millennials (also known as Generation Y) (1981–1996) are characterised by the desire for meaningful work, the opportunities for continuous learning and stronger preference for work–life balance. Generation Z (1997–2010), the youngest group of doctors currently in our workforce, places even more importance on flexibility and social responsibility, expecting employers to offer not only good working conditions, but also to take stances on broader social, political and employment issues [19]. With a shifting focus to satisfy the priorities of generation Z, employers in 2020 reported a move toward a better working environment and work–life balance.

Although not by any means universal, the differences observed between the generations of doctors’ working preferences in our study, therefore, is perhaps unsurprising. In addition to this, during the last 5 years of practice, there has been significant tumult in burns care worldwide following from the COVID-19 pandemic. The working conditions before and after 2020 are within the working memories of doctors of Millennial and older generations; however, they are not for generation Z. Most have negative reflections on this period and its ongoing impact on working conditions and the provision of burns care itself, when compared to the period before. This may be a further contributing factor for the generational skew in voting preferences observed.

There are limitations with this survey. The recollections are of over half a century ago, and are those of a single resident doctor, working in a single burns centre for a short period, and thus provide a single snapshot. This survey was similarly from a self-selecting group of interested parties within the same unit, each with their own varied levels of experience as a burns doctor. Finally, the numbers in this survey are relatively small, thus preventing the application of more powerful statistical tests such as binary logistic regression. Larger sample sizes would be required for more conclusive results.

A living link to a pioneering burns surgeon led to many local discussions about the huge changes that have taken place in burns care in the last half century, and how proud and positive we can be about the future of burns surgery as a career. It also highlighted the changing views of the workforce, and whilst not designed to be a comprehensive social study, again provided useful insights for future planning of jobs within the specialty. Many of the changes are causes for celebration within the burns community: the improved survival rates due to pioneers such as Douglas Jackson; the decrease in flame burns and industrial injury through better legislation; the better work–life balance now, meaning a job in burns surgery is open to more trainees; opportunities in research and enthusiastic teamwork mean that burns surgery is still a career with a bright future.

## 6. Summary

Wide-ranging differences in the domains of finance, working patterns and clinical care have been described in the same burns unit 50 years apart. When presented with this objective data, the majority of clinicians working in the same department today feel the working conditions at present are preferential. It is noticeable, however, that this was not universal, with inter-generational differences demonstrated. We invite you to reflect on your current or past experience working as a resident burns doctor, and consider when you would rather have worked.

## Figures and Tables

**Table 1:** Summary of information presented to those participating in survey (* 10-year review 2014–2024 Birmingham, unpublished data).

Life/Work Domain		1970	2024
**Working pattern**	Starting the morning	9 a.m. ward round, beginning with prayer	8 a.m. ward round. 07:30 a.m. Wednesday
	Finishing	Dictated by work load—3 p.m.–7 p.m.	5 p.m. regardless of workload
	On call/hours	Alternate nights and alternate weekends (1:2) Average 113 h a week	7 nights in 4 months 1 weekend per month 1 on call week every 2 months Off days either side of on calls, average 48 h a week
	Number of beds	30	15 +/− 2 ITU
	Pagers	Individual pager	Pager only when on call
	Staffing	2 consultants, 2 SHOs	4 registrars, 3–4 SHOs, 7 consultants
**Finances**	Basic salary (2024 equivalent)	GBP 23,643 per annum	GBP 37,303 per annum
	Accommodation	GBP 0 per annum	Rent/mortgage and household bills roughly GBP 14,000–18,000 per annum
	Living Expenses	All meals free, nurses would often bring doctors sandwiches	Privatised catering. GBP 4.50 staff offer per meal from the canteen
	Spare time	Restricted. Doctor’s mess main source of entertainment	Most evenings and weekends
**Burns care**	Dressings	Consultant- and Doctor-delivered Silver nitrate solution or silver sulfadiazine Changed every day	Nurse-led Variety of dressing options Clinical photos taken Changed usually every 48–72 h
	Outcomes	>70% TBSA then commonly un-survivable	Baux score 140 considered survivable
	Resus burns	>17% TBSA. Hourly adjusted fluids, catheter, hematocrit measured hourly by SHO by hand	>15% TBSA, Fluid resus using Parklands formula and goal-directed therapy, arrive from midlands via air or ambulance into ED
	Elective scar procedures	None—not many cases taken to theatre	Multiple-scar prevention with conservative measures, Pulse Dye and Fractionated CO_2_ laser, steroid injections, fat grafting, scar revision and resurfacing
	Skin grafting	<1 inch left to heal >1-inch graft-taken with Braithwaite knife, drum dermatome, meshed then dressed. Graft check at 24 h to milk haematoma and readjust graft. Excess graft banked in skin fridge for further ward-based application	Early tangential excision for deep dermal and full thickness burns. Air-driven dermatome and meshed split skin graft. Use of dermal substitutes
	Burn aetiology	Heating appliances 38% Flash flame (gas/fireworks) 24% Scalds 14% Molten material 8% Other (electrical) 16%	Scald 25% * Flame 22% * Flash flame 15% * Contact 9% * Chemical 7% * Other/not stated 22% *

**Table 2:** Retail price index (“RPI”—a measure of inflation measured by the UK’s office for national statistics) adjusted doctors’ salary in 1970.

Grade of Doctor	Average Basic Salary 1970 (GBP PA)	RPI Adjusted Salary to 2024 (GBP PA)	Actual Basic Salary 2023/2024 (GBP PA)
House officer (year 1)	1450	19,152	32,398
Senior House officer (year 2)	1790	23,643	37,303
Registrar	2220	29,323	43,923–55,329
Senior Registrar	2760	36,456	63,152
Consultant	9275	122,512	93,666–126,281

## Data Availability

The original contributions presented in this study are included in this article; further inquiries can be directed to the corresponding author.
